# RNAi Targeting of West Nile Virus in Mosquito Midguts Promotes Virus Diversification

**DOI:** 10.1371/journal.ppat.1000502

**Published:** 2009-07-03

**Authors:** Doug E. Brackney, Jennifer E. Beane, Gregory D. Ebel

**Affiliations:** 1 Department of Pathology, University of New Mexico School of Medicine, Albuquerque, New Mexico, United States of America; 2 The Pulmonary Center, Boston University Medical Center, Boston, Massachusetts, United States of America; The Pennsylvania State University, United States of America

## Abstract

West Nile virus (WNV) exists in nature as a genetically diverse population of competing genomes. This high genetic diversity and concomitant adaptive plasticity has facilitated the rapid adaptation of WNV to North American transmission cycles and contributed to its explosive spread throughout the New World. WNV is maintained in nature in a transmission cycle between mosquitoes and birds, with intrahost genetic diversity highest in mosquitoes. The mechanistic basis for this increase in genetic diversity in mosquitoes is poorly understood. To determine whether the high mutational diversity of WNV in mosquitoes is driven by RNA interference (RNAi), we characterized the RNAi response to WNV in the midguts of orally exposed *Culex pipiens quinquefasciatus* using high-throughput, massively parallel sequencing and estimated viral genetic diversity. Our data demonstrate that WNV infection in orally exposed vector mosquitoes induces the RNAi pathway and that regions of the WNV genome that are more intensely targeted by RNAi are more likely to contain point mutations compared to weakly targeted regions. These results suggest that, under natural conditions, positive selection of WNV within mosquitoes is stronger in regions highly targeted by the host RNAi response. Further, they provide a mechanistic basis for the relative importance of mosquitoes in driving WNV diversification.

## Author Summary

West Nile virus (WNV) was introduced into New York state in 1999 and has since spread across the Americas. It is transmitted in nature between adult female mosquitoes and birds and occasionally infects humans and horses. Within the host, WNV exists as a diverse assortment of closely related mutants. WNV populations within mosquitoes are more complex genetically than are those within birds. The reasons for this discrepancy are unknown, but may be related to the host's innate antivirus response. We demonstrate that WNV is targeted by RNA interference, a highly sequence-specific pathway in the mosquito. Further, we present data that correlates the intensity of this targeting with virus mutation under natural conditions. These results provide a mechanistic explanation for the increasead complexity of WNV populations in mosquitoes: the RNAi response creates an intracellular environment where rare genotypes are favored. In addition, our results suggest that genetically diverse WNV populations may have an advantage over less diverse populations because they present a more complex target for the RNAi response. Finally, these data suggest that WNV, and possibly other viruses with high mutation rates, may escape an engineered antivirus intervention that is highly sequence-specific.

## Introduction

RNA viruses possess an extraordinary capacity to adapt to changing environments due to their high mutation rate [1]. Recent epidemics of West Nile virus (WNV, *Flaviviridae*, *Flavivirus*) and chikungunya virus (CHIKV, *Togaviridae*, *Alphavirus*) were driven by relatively small genetic changes that increased the efficiency with which the viruses were transmitted by mosquito vectors (*Culex spp.* and *Aedes albopictus*, respectively) [2]–[4]. In the case of WNV, a single conservative amino acid substitution is responsible for increased transmission efficiency at early timepoints after the mosquito acquires an infectious bloodmeal [2],[3]. The change in transmission efficiency has led to the complete displacement of the parental WNV genotype by the derived strain, demonstrating the power of small genetic changes to profoundly influence arbovirus transmission patterns and the importance of mosquitoes in shaping their populations [5]. Studies examining the population genetics of WNV have shown that WNV consists of a genetically diverse population within hosts, and that infections within mosquitoes are more genetically diverse than are those within birds [6]. The underlying mechanism for the increased genetic diversity in mosquitoes, however, is poorly understood.

Eukaryotic organisms generally possess pathways that respond to pathogen-associated molecular patterns (PAMPs) that, when activated, lead to suppression or elimination of the pathogen. Double-stranded RNA (dsRNA) is one such PAMP that is a powerful trigger of innate antiviral responses. Invertebrates, including mosquitoes and other dipterans, respond to virus infections through RNA interference (RNAi) [7]–[13]. In RNAi, dsRNA molecules are identified, processed and ultimately used to guide sequence-specific degradation of homologous RNA sequences. Because many RNA viruses, including WNV and other arboviruses, have complex secondary structures within their genomes and form dsRNA intermediates during replication, their genomes may be susceptible to RNAi-based degradation. Indeed, several studies have demonstrated that the RNAi pathway can function to limit viral infections in members of the diptera [7]–[12]. Virus-derived small interfering RNAs (viRNAs) have been detected in *Aedes aegypti* mosquitoes infected with the arboviruses dengue virus (DENV) and Sindbis virus (SINV), and in *Drosophila* infected with flock house virus (FHV) [7],[9],[10]. In addition, suppression of RNAi machinery in *Ae. aegypti* resulted in transient increases in DENV and SINV titers [10],[14]. Similar studies in RNAi deficient *Anopheles gambiae* showed increased viral dissemination rates and titers of intra-thoracically inoculated O'nyong-nyong virus [15]. An expanding body of literature thus suggests that RNAi functions as an innate antivirus response in mosquitoes. Interpreting this literature, however, has been problematic. First, the systems studied have generally not included ecologically relevant virus-vector pairs (but with the notable exception of Sanchez-Vargas et al. [10]). For example, neither *Aedes aegypti* nor *Drosophila* are vectors of SINV or FHV, respectively. Second, although mosquitoes acquire infection orally during bloodfeeding, many published studies have examined intra-thoracically inoculated mosquitoes. Mosquito midgut epithelial cells are the first cells to become infected and midgut tissues present important infection and escape barriers that appear to control the vector competence of mosquitoes for arboviruses [16]–[20]. Consequently, surprisingly little is known about the innate antivirus response mounted by ecologically important vector mosquitoes in physiologically relevant tissues.

Viruses possess counter-measures to escape host antiviral responses. One such counter-measure may be their characteristically high mutation rates. RNAi is highly sequence specific and single nucleotide mismatches between the guide and target sequences can drastically reduce or abolish silencing effects [21],[22]. The sequence specificity of the RNAi response may therefore influence virus genetics. For example, artificial induction of the RNAi pathway using virus-specific siRNAs has been shown to result in (a) the accumulation of mutations in the targeted regions and (b) viral escape in poliovirus (*Picornaviridae*; *Enterovirus*), hepatitis C virus (*Flaviviridae*; *Hepacivirus*), LaCrosse virus (*Bunyaviridae*; *Bunyavirus*) and HIV-1 [23]–[26]. Whether this occurs spontaneously following oral infection of competent vector mosquitoes, however, is less clear. Since WNV genetic diversity is increased within mosquitoes, we hypothesized that RNAi might lead to increased mutational diversity by creating an intracellular environment in which mutant genomes are less likely to be degraded by viRNAs deriving from common WNV genome sequences. In particular, we used a deep sequencing approach (technology reviewed extensively in [27]) to determine whether siRNAs mapping to the WNV genome are produced in mosquito midguts in response to infection. Using these data, we then determined whether particular genome regions are consistently targeted and whether differential targeting of the genome leads to increases in genetic diversity in intensely targeted regions.

## Results

### Mosquito infection

To ensure that only WNV-positive midguts were included in the infected group, and that all putatively negative mosquito midguts were indeed negative, midgut RNAs were screened for the presence of WNV RNA by RT-PCR. At both timepoints sampled, 12 of the 15 midguts analyzed were positive, and all control mosquito midguts were negative.

### Characterization of viRNAs from *Cx. p. quinquefasciatus* midguts

**Figure 1:**
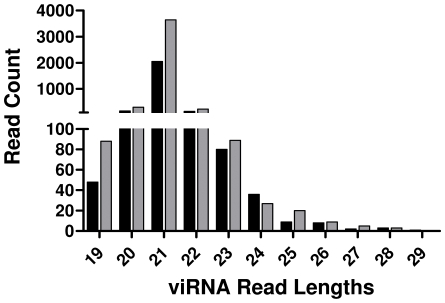
Size and Abundance of Small RNA Reads Mapping to the WNV Genome. The total abundance of sRNAs mapping to the WNV genome based on sRNA length. Black and grey bars correspond to 7 and 14 days post infection, respectively.

To define the population of small RNAs in mosquito midguts, small RNA (sRNA) libraries from WNV-infected and uninfected midguts after a 7 or 14 day extrinsic incubation period (EIP) were subjected to high-throughput sequencing. The total number of sRNA reads obtained from each of the four libraries sequenced ranged from 3.7 to 4.9 million (Table 1). To determine the presence of viRNAs, sRNA reads were aligned to the WNV genome. There were 2,544 (1,701 unique) and 4,419 (2,629 unique) reads that aligned to the WNV genome following a 7 or 14 day EIP, respectively, where unique reads represent individual viRNAs that code for a specific nucleotide sequence. As expected, these reads had a mean and mode length of 21 nt (Figure 1, Table 1). In addition, the average quality scores [Illumina scores, scaled from 1 (minimum) to 40 (maximum)] were extremely high (90% of the reads >36) and greater than 80% of these reads perfectly aligned with the viral genome (Table 1). A small population of sRNAs from the uninfected mosquito midguts aligned to the WNV genome. A high proportion of these reads required 2 mismatches in order to align to the WNV genome; 50% and 86% at 7 and 14 days post bloodmeal, respectively, (Table 1).

**Table 1:** Characteristics of small RNA libraries sequenced.

Mosquito sRNA library sequenced:	Total # reads (×10^6^)	Reads Aligning to WNV genome:			Number of mismatches required for alignment n (%):		
		Total # (# unique)	Average Length (nt)	Average Quality Score	0	1	2
WNV (−) 7 dpi	4.9	18 (14)	20.6	37.8	7 (39)	2 (11)	9 (50)
WNV (−) 14 dpi	3.9	65 (21)	20.5	38.9	8 (12)	1 (2)	56 (86)
WNV(+) 7 dpi	4.7	2,544 (1,701)	21.1	38.5	2,104 (83)	343 (13)	97 (4)
WNV(+) 14 dpi	3.7	4,419 (2,629)	21.0	38.8	3,754 (84)	513 (12)	152 (3)

The orientation of the WNV strand targeted by viRNAs was determined. At both a 7 and 14 day EIP, 74% of all viRNAs were derived from the positive sense viral genome. Subsequently, genomic equivalents for both the positive and negative strands were determined by two-step RT-PCR. 81% and 80% of the total WNV RNA detected was from the positive strand at 7 and 14 days, respectively.

### viRNA distribution and abundance

The percent coverage of the WNV genome by viRNA was determined. 81.75% and 91.88% of the genome was targeted by at least one viRNA in the WNV infected samples following a 7 or 14 day EIP, respectively.

**Figure 2:**
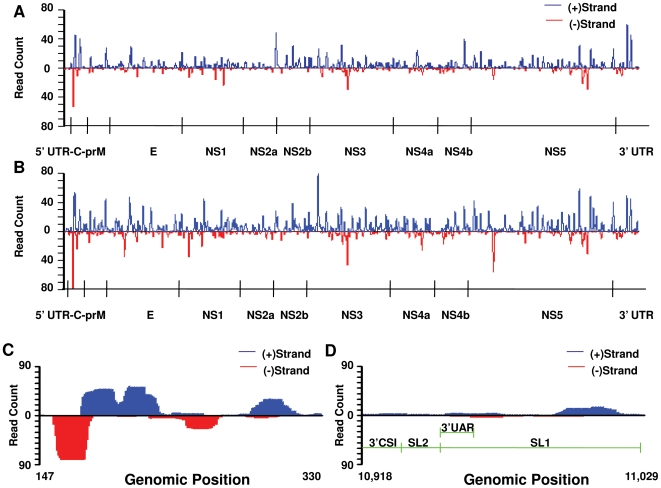
viRNA Coverage of WNV Genome at 7 and 14 Days Post Infection. Complete genome of WNV showing the intensity of viRNA coverage at each nucleotide of the genome at 7 (A) and 14 (B) days postfeeding. Reads originating from the positive strand are shown in blue, above the axis, and those originating from the negative strand are shown in red, below the axis. Particular regions appear to be preferentially targeted by the RNAi response, including the 5′ aspect of the C coding region and the 3′ non-coding region. Peaks are generally higher at 14 than 7 days post infection (dpi), with notable exceptions in NS2a, at the NS4b/NS5 junction and the 3′-UTR. Both positive and negative polarity RNA is targeted by viRNAs generated in mosquito midgut cells. At 14 dpi, although true for both 7 and 14 dpi, the focus of viRNA targeting in the C coding sequence (C) is characterized by a relatively intense targeting of the negative strand compared to the 3′-UTR (D). Notably, well documented imperfect stem-loops and other critical functional RNA structures within the 3′-UTR do not appear to be intensely targeted by the RNAi response.

To assess positional and regional differences in the intensity of viRNA targeting of the WNV genome in mosquito midguts, the frequency of viRNA reads mapping to each nucleotide in the WNV genome was computed (Figure 2A). Inspection of these results revealed (A) an asymmetric distribution of viRNAs across the genome with some regions being highly targeted and others weakly or not targeted and (B) that peaks in the frequency distribution of hits along the genome at 7 days were also apparent at 14 days, although peaks at 14 days tended to be higher, with some exceptions.

The most intensely targeted portion of the WNV genome, at both 7 and 14 days, was an approximately 200 nt region of the Capsid (C) coding sequence. Genome position 176 within this region was targeted by 64 and 120 viRNAs at 7 and 14 days, which was the highest peak in either dataset. Examination of the orientation of viRNAs targeting this region revealed roughly equal targeting of both positive and negative strand WNV genomes (Figure 2C). A similar examination of a portion of the 3′UTR that possesses well-characterized secondary structures revealed less intense targeting, and a more pronounced bias toward viRNAs targeting the positive WNV genome (Figure 2D).

**Figure 3:**
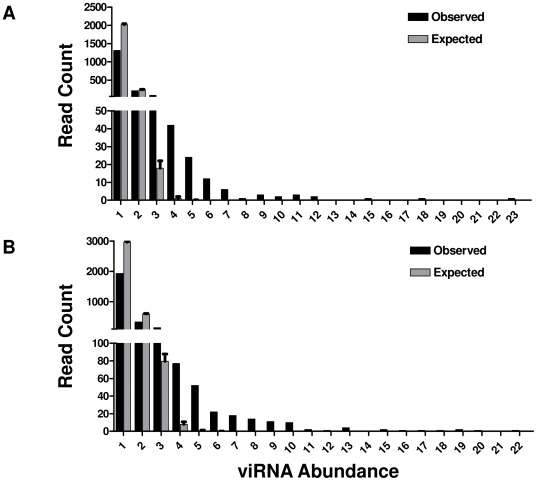
A Small Subset of viRNA Reads Is Highly Abundant. viRNA abundance observed at 7 (A) and 14 (B) days post infection (black bars) compared to the null expectation of equal abundance of all reads (grey bars). Expected values were obtained through permutation analysis with replacement sampling of n = 2544 (day 7) or n = 4419 (day 14) random genome positions. 100,000 permutations were conducted for each dataset. Expected and observed distributions of read abundances were statistically compared by the Kolmogorov-Smirnov test (P<0.001 at both timepoints).

**Figure 4:**
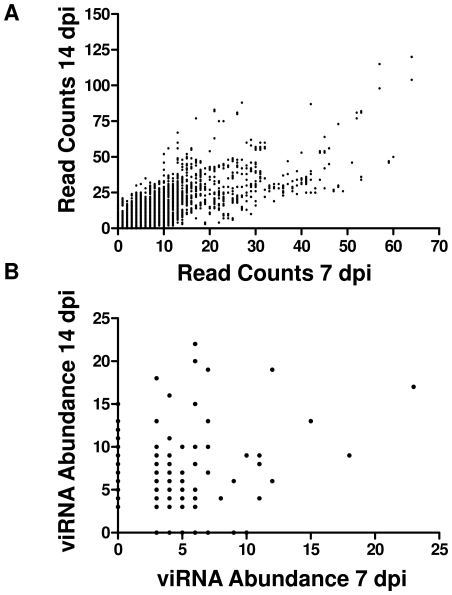
Targeting of the WNV Genome by RNAi at 7 and 14 Days Post Infection Is Correlated. (A) Count of reads aligning to each genome position at 7 and 14 days, n = 11,029, Spearman r = 0.7610, p<0.0001. (B) Count of abundant individual sequence reads in libraries obtained at 7 and 14 days, n = 433, Pearson R-squared = 0.03674, p<0.0001.

We then examined viRNAs that were apparently abundantly produced in mosquito midguts. At both 7 and 14 days the abundance of each unique read was calculated which ranged from 1 to 23 (Table 1). In order to assess whether the observed distribution of read abundance at both 7 and 14 days was significantly different than would be expected by chance, we conducted a permutation analysis. For each time point, n = 2544 (day 7) or n = 4419 (day 14) genomic positions were randomly sampled with replacement. The sampling procedure was repeated 100,000 times to obtain a theoretical distribution of read abundances, and this distribution was compared to the observed distribution by the Kolmorogov-Smirnov test (Figure 3). The observed abundance distribution was significantly different from the null expectation (P<0.001 at 7 and 14 days). In addition, the intensity of viRNA targeting of each nucleotide of the genome was highly correlated at 7 and 14 days (Figure 4A). Further, a subset of viRNAs was detected at both sampled timepoints. The abundance of these “common” reads at 7 and 14 days was also highly correlated (Figure 4B).

### RNAi influence on virus diversification

The RNAi pathway is highly sequence specific and mutations in the target dsRNA sequence can have profound effects on its efficiency. Therefore we sought to determine whether highly targeted regions would be more likely to contain mutations compared with relatively weakly targeted regions. Initially, we used imperfect reads (i.e. reads that required one or two mismatches in order to be aligned to the WNV genome) as a proxy for viral mutant sequences, hypothesizing that these reads derived from mutant genomes and had been processed by the host cell RNAi machinery. Putative mutant basecalls with quality scores of less than 30 were discarded. Thus, the probability that basecalls included as mutations in this analysis are read errors was less than 0.0010. At both timepoints sampled, mismatched nucleotides within imperfect reads were covered by a significantly higher number of viRNAs compared to nucleotides that had only perfect reads and were covered by at least a single viRNA (Table 2, T-test P<0.0001).

**Table 2:** Increased viRNA coverage of WNV genome at imperfectly aligned positions.

Match type:	Mean viRNA coverage per nt (Std. Dev., n)	
	7 dpi	14 dpi
Perfect	5.80 (7.00, 8,707)	8.91 (9.96, 9,712)
Imperfect	10.72 (10.76, 300)	15.40 (14.32, 420)
P-value*	6.14×10^−14^	1.63×10^−18^

*: T-test for independent samples with unequal variances.

**Figure 5:**
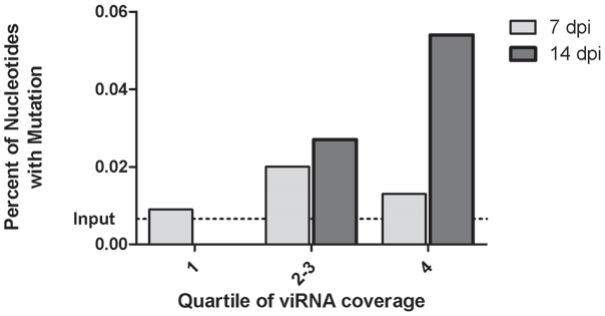
Intense Targeting of WNV Genome Is Associated with High Mutational Diversity. viRNA coverage intensity was classified according to quartiles. Interquartile ranges (min, max) at 7 and 14 days were 1–6 (0, 64) and 3–11 (0, 120), respectively. The total number of nucleotides sequenced in each coverage class was 10,225, 5,062 and 7,475 at 7 dpi and 5,990, 7,442 and 7,438 at 14 dpi. Statistical significance was determined by chi squared test for trend. A significant association between viRNA coverage was detected at 14 dpi (p = 0.0393) but not at 7 dpi (p = 0.8107).

Next, we tested the association between viRNA coverage and mutation frequency directly. To accomplish this, we identified regions in NS5 and the 3′-UTR of the WNV genome that had varying degrees of viRNA coverage and sampled WNV genomes from the same RNA specimens used to generate sRNA libraries. viRNA coverage per nucleotide along the entire genome was used to determine the frequency distribution of number of viRNA hits per nucleotide position. Positions were then ranked into quartiles and the interquartile range combined. At 7 days there were three mutations identified in NS5 and no mutations in the 3′-UTR. These mutations were evenly distributed across the three frequency distribution classifications. In WNV genomes sampled at 14 days, mutations were detected in both NS5 and 3′-UTR with increasing viRNA coverage associated with increasing mutation frequency. (Chi-squared test for trend, p = 0.0393) (Figure 5).

## Discussion

We used massively parallel sequencing to comprehensively analyze viRNA from the midgut of *Cx. p. quinquefasciatus*. Mosquitoes were sampled after a 7 or 14 day EIP in order to assess differential production and positioning of viRNAs aligned to the WNV genome. At both sampling points, 80% of midguts were infected, indicating that the *Cx. p. quinquefasciatus* used in our studies were highly susceptible to infection by WNV. WNV-positive midgut RNA samples from each were then pooled in order to obtain sufficient RNA concentrations for sRNA library construction. In addition, pooling RNA allowed us to look at a generalized response to WNV within midguts. Additional studies will be required to characterize individual mosquito RNAi responses to WNV.

Between two and five thousand high-quality reads from infected midguts mapped to the WNV genome at both sampling points (Table 1). The average read length was approximately 21 nt, and the vast majority were between 20 and 22 nt, suggesting that reads mapping to the WNV genome were mainly viRNAs (Figure 1). Furthermore, the majority of these reads matched perfectly to the sequence of the infectious clone-derived WNV used to infect the mosquitoes. In contrast, a very small population (<70 reads) of siRNAs mapping to the WNV genome were identified from uninfected control mosquitoes. These reads were similar in length and quality to those from infected midguts, but tended to require more mismatches to align to WNV (Table 1). The origin of these siRNAs is unknown, although the presence of insect-specific flaviviruses such as *Culex* flavivirus and T'Ho virus has been reported in wild *Culex spp.* populations and could have led to the observed data [28]–[31]. In addition, flavivirus-like genetic material, termed cell-fusing agent virus, has been identified from the genomes of *Culex spp.* populations in Puerto Rico [32]. To eliminate the possibility that these viruses were the source of the siRNAs in our uninfected control mosquitoes, three pools of ten male and female *Cx. p. quinquefasciatus* from our colony were screened for the presence of flavivirus RNA by one-step RT-PCR using flavivirus specific primers [28]. By this approach, we were unable to detect the presence of flavivirus RNA in our colony mosquitoes, suggesting that these siRNAs were not derived from a contaminating insect-specific flavivirus (data not shown). Further, alignment of our sRNA libraries against multiple irrelevant *Flavivirus* genomes and one *Alphavirus* genome produced very few reads mapping to anything other than WNV (Text S1, Table S2). Alternatively, there may have been cross contamination between the infected and uninfected samples during preparation of the sRNA libraries. However, this seems unlikely considering that the majority of the siRNAs identified in the uninfected control groups contained mismatches (61% and 88% at 7 and 14 days post bloodmeal, respectively) while the viRNAs retrieved from the infected samples were predominantly perfect matches (83% and 84% at 7 and 14 days post infection, respectively) with the WNV genome (Table 1). Finally, because the genome of *Culex pipiens* has not been completed we cannot rule out the possibility that these sequences were derived from the *Culex* genome. In any case, examination of basic quality metrics on read data from all four sRNA libraries allows us to conclude that the viRNA sequences obtained from infected midguts are of generally high quality and are products of the RNAi pathway.

We observed viRNAs targeting both the positive and negative sense WNV genome. *Flaviviruses* have highly structured genomes with many secondary structures and replicate in an asymmetric manner through dsRNA replicative intermediates. While dsRNA clearly activates the RNAi pathway, it has not been clear whether replicative intermediate or stem-loop and other RNA secondary structures are mainly responsible for activation. Studies with (+) ssRNA plant viruses have demonstrated that either may predominate, depending on the virus-host system studied. For example, infection of two different plant species with *Cymbidium ringspot tombusvirus* resulted in the production of viRNAs almost exclusively from the positive-strand. These results suggest that imperfect duplexes in the secondary structure of the (+) ssRNA genome serve as targets for Dicer (DCR) cleavage [33],[34]. Conversely, when analyzing the viRNA profile of *Brassica juncea* infected with *Turnip mosaic potyvirus*, viRNAs originated from both the positive- and negative-strands and were present in almost equal proportions suggesting that DCR targets dsRNA replicative intermediates in this system [33]. We found that RNAi targeting of the negative sense genome was proportional to the amount of negative sense genome in the infected midguts, which was also observed in the FHV-drosophila system [7]. In addition, a highly structured portion of the 3′UTR was not highly targeted in our data. These results differ from those recently reported by Myles and others whom reported a strong positive-strand bias in *Aedes aegypti* inoculated with SINV [9]. The reasons for this are likely to be complex, and possibly related to differences in the virus-vector system, route of infection and/or source of sRNAs (midguts vs. whole body), among others. In any case, our data establish that the RNAi pathway in WNV-infected *Culex* midguts clearly targets dsRNA replicative intermediates and most likely secondary structures within the positive-sense genomic RNA as well.

Computing the number of viRNA reads targeting each nucleotide of the WNV genome allowed us to determine whether particular regions are more intensely targeted by the RNAi response than others. Inspection of Figure 2 clearly shows that this is true. Further, regions targeted at 7 days were also targeted at 14 days: peaks in the figure tend to coincide with one another. Indeed, the per-nucleotide intensity of viRNA coverage along the genome at 7 and 14 days is highly correlated (Figure 4A). This suggests that particular regions of the genome are more accessible to the RNAi pathway than others. Supporting this observation, some peaks are extremely high, while others are quite small. Notably, the most intense targeting of WNV by viRNAs at both timepoints is a region in the 5′ aspect of the C coding region (Figure 2C). The reasons for this are unclear at present, but may be related to inefficient replication initiation, whereby a stalled replicase complex may leave dsRNA targets available for cleavage. In fact, this has been described in *Drosophila* using FHV [7]. Interestingly, the extreme 3′ portion of the 3′UTR, which has well characterized stem-loop structures, is not highly targeted (Figure 2D). Upon further analysis of the distribution of viRNA aligning to the WNV genome a small number of sequences obtained from both infected libraries were detected very frequently. The possibility that this observation was due to a sampling artifact was ruled out by permutation analysis. This suggests that detecting any read greater than three times was unlikely to occur by chance (Figure 3). Therefore, we considered any viRNA with abundance greater than three to be highly abundant. We also observed that the abundance of read sequences detected at both 7 and 14 days (i.e. “common reads”) were highly correlated (Figure 4B). Collectively, these data demonstrate that there are ‘hotspots’ within the WNV genome targeted by the RNAi pathway in the midgut of *Cx. p. quinquefasciatus* and suggest a stereotypical RNAi response to WNV infection that is characterized by the production of a small subset of common viRNA molecules.

WNV populations within hosts are highly genetically diverse, with greater levels of population diversity (i.e. more mutations) found in mosquitoes compared to birds [6]. The mechanism(s) that give rise to this increase in genetic diversity within mosquitoes, however, remain poorly characterized. We hypothesized that the sequence specificity of the RNAi response in mosquitoes might increase viral genetic diversity. Mutant genomes would be favored because they would be less susceptible to degradation by the RISC loaded with wild-type, un-mutated viRNAs, i.e., they could escape degradation by mutation. We observed two lines of evidence in support of this. First, viRNAs that were imperfect matches to the input WNV genome tended to occur on nucleotides that were significantly more highly targeted than un-mutated nucleotides, excluding uncovered positions (Table 2). Second, when viral genetic diversity was independently assessed using viral RNA from the midguts analyzed, it was found that after fourteen days extrinsic incubation, regions that were more intensely targeted by viRNAs had greater genetic diversity than regions that were weakly targeted (Figure 5). To ascertain whether the observed results were a result of differences in selective constraints across the genome, we analyzed the selective pressures influencing the WNV populations within the mosquito midgut (Text S1, Figure S1, Table S1). These analyses failed to detect significant correlation between intrahost mutational diversity (estimated from sRNA reads mapping to the WNV genome) and dN/dS or interhost genetic diversity. Thus, our data support the hypothesis that intense targeting of WNV by the RNAi pathway in mosquitoes might result in the observed increases in genetic diversity in mosquitoes relative to birds. Indeed, this phenomenon has been observed by other investigators. Multiple studies have demonstrated that artificial induction of the RNAi pathway with viral-specific siRNAs can drive viral evolution [22],[25],[26]. For example, exposure of HIV-1 to viral-specific siRNAs resulted in the accumulation of mutations and viral escape mutants. When the viral escape mutants were sequenced it was found that they contained a mutation distribution similar to sequence variants of naturally circulating HIV-1 [23]. It is notable that even with the power constraints implicit in our strategy for independently assessing genetic diversity in our samples (a relatively small number of nucleotides were sequenced) we observed a significant association between viRNA coverage and viral genetic diversity. High-throughput virus genome sequencing would undoubtedly enhance our ability to characterize the WNV genotypes in our samples. Nonetheless, our results on viral genetic diversity describe for the first time a direct correlation between the RNAi pathway and viral evolution under natural conditions. Further, they suggest a mechanism for the increased fitness observed in highly genetically diverse WNV populations *in vitro* [35] and may in part explain how WNV can persist *in vivo* in mosquitoes despite the presence of an apparently robust RNAi response. A genetically diverse virus population, whether acquired during bloodfeeding or arising through mutation *in situ*, may present a more complex target for the RNAi response.

## Materials and Methods

### Virus

WNV used in these experiments was generated from an infectious cDNA clone derived from the NY99 strain as previously described [36]. Virus was produced in baby hamster kidney (BHK) cells and used without subsequent passage. WNV obtained in this manner is highly genetically homogeneous [6], and well characterized phenotypically [37].

### Mosquitoes

The mosquitoes used for these experiments were obtained from our *Culex pipiens quinquefasciatus* colony. Mosquitoes were housed in environmental chamber at constant temperature of 27°C with a 16∶8 light∶dark photoperiod for the duration of these experiments. Artificial bloodmeals containing defibrinated goose blood alone (controls) or with 2×10^8^ plaque-forming units (pfu)/ml of WNV were offered to adult female *Cx. quinquefasciatus* five to seven days post-eclosion using a Hemotek (Accrington, UK) membrane feeding apparatus. Mosquitoes were then cold anesthetized and engorged individuals reserved and held for either a 7 or 14 day EIP under the above standard conditions. After the 7 and 14 day EIPs, mosquitoes were cold-anesthetized and fifteen mosquitoes from each group were dissected. Midguts were isolated, washed four times in phosphate buffered saline (PBS) and placed in 300 µl lysis buffer (mirVana, Ambion, Austin, TX). Forceps were flame sterilized between each dissection.

### RNA extractions and one-step reverse transcriptase polymerase chain reaction (RT-PCR)

RNA from individual midguts was extracted using the mirVana miRNA Isolation Kit according to the manufacturer's instructions. The midgut RNA was subsequently analyzed by one-step RT-PCR for the presence of WNV RNA using the SuperScript® One-Step RT-PCR with Platinum® Taq (Invitrogen, Carlsbad, CA) according to standard methods. WNV-positive midgut RNAs, were pooled. An equivalent number of uninfected midgut RNAs were pooled in our control groups. RNA was then precipitated with ethanol, resuspended in 100 ul H_2_O, and quantity and integrity determined on an Agilent 2100 Bioanalyzer (Agilent Technologies, Santa Clara, CA).

### Preparation of small RNA populations and high-throughput sequencing

Approximately 6–7 µg of total RNA from pooled WNV-positive and negative specimens were size fractionated on a 15% TBE/ urea polyacrylamide gel and small RNA (sRNA) populations (18–30 nt) recovered. 5′ and 3′ sequencing adapters were then ligated to sRNAs and reverse transcription and PCR amplification performed according the manufacturer's instructions (Illumina, San Diego, CA). The resulting libraries were sequenced at the National Center for Genomic Resources (Santa Fe, NM) using an Illumina Cluster Station and Genome Analyzer.

### Assembly and analysis of sRNA libraries

Reads from small RNA libraries were trimmed of adapter sequences and aligned to the WNV infectious clone reference genome using the Short Oligonucleotide Alignment Package v.1 (SOAP) (http://soap.genomics.org.cn/) with seed size of eight, and a maximum of two mismatches allowed. Further trimming and gaps were not permitted. Additional analyses were performed using R, Microsoft Excel and GraphPad.

### Determination of WNV genetic diversity

Intra-population genetic diversity was determined according to methods described elsewhere [6]. For these studies, 160 nt (10776–10936) and 454 nt (7800–8254) of the 3′-UTR and NS5 of the WNV genome, respectively, were chosen based on inspection of frequency distributions of the number of viRNA “hits” per nucleotide. Regions were chosen that had highly variable viRNA coverage. Viral RNAs extracted from WNV-infected pools were reverse transcribed using the AccuScript High Fidelity 1^st^ Strand cDNA Synthesis Kit (Stratagene, LaJolla, CA). The fragments were PCR amplified using high fidelity *Pfu*Ultra polymerase (Stratagene) using the following parameters: 94°C for 30 s, 60°C for 30 s and 72°C for 45 s repeated an additional 39 cycles followed by a 72°C final extension for 6 min. The primers used for both the RT and PCR reactions were WNV 10,524 F 5′- CGCCACCGGAAGTTGAGTAGAC -3′ and WNV 3′ End 5′- AGATCCTGTGTTCTCGCACCACCA - 3′ or WNV 7670 F 5′-GACTAAAAAGAGGTGGGGCAAAAG -3′ and WNV 8335 R 5′-GAAGCTCGACTCACCCAATACAT -3′. Amplicons were gel purified and cloned into pCR Script Amp^+^ vector (Stratagene). Clones were sequenced using the M13 Rev primer at the University of New Mexico's School of Medicine DNA Services laboratory. Sequences were aligned and analyzed for genetic diversity using DNAStar's SeqMan program.

### Quantitative reverse transcriptase polymerase chain reaction (Q-RT-PCR)

To determine the amount of positive and negative strand WNV genome in mosquito midguts at each sampling point, equal concentrations of RNA from WNV-infected midguts was reverse transcribed with strand specific primers using the AccuScript High Fidelity 1^st^ Strand cDNA Synthesis Kit (Stratagene). WNV RNA of each polarity was then quantified using real-time quantitative (TaqMan) RT-PCR as described elsewhere [38].

## Supporting Information

Figure S1 Intrahost mutational diversity and dN/dS, Pi and viRNA coverage.
(1.73 MB TIF)

Table S1 Genetic diversity and selection in cloned WNV. * An alignment of 38 WNV genomes obtained from the North American epidemic was used to generate interhost diversity values. # Undefined. Only nonsynonymous mutations were present in the alignment, yielding a denominator of zero for dN/dS calculation.
(0.01 MB PDF)

Table S2 Alignment of sRNA reads against viruses not used in these studies. * For all four sRNA libraries generated in these studies (WNV-infected and uninfected).
(0.01 MB PDF)

Text S1 Supplemental text.
(0.04 MB PDF)
