# Supplementary figures and images for: RNAi Targeting of West Nile Virus in Mosquito Midguts Promotes Virus Diversification
Source: PLoS Pathog. 2009 Jul 3;5(7):e1000502. doi: 10.1371/journal.ppat.1000502 (PMC2698148; doi:10.1371/journal.ppat.1000502)

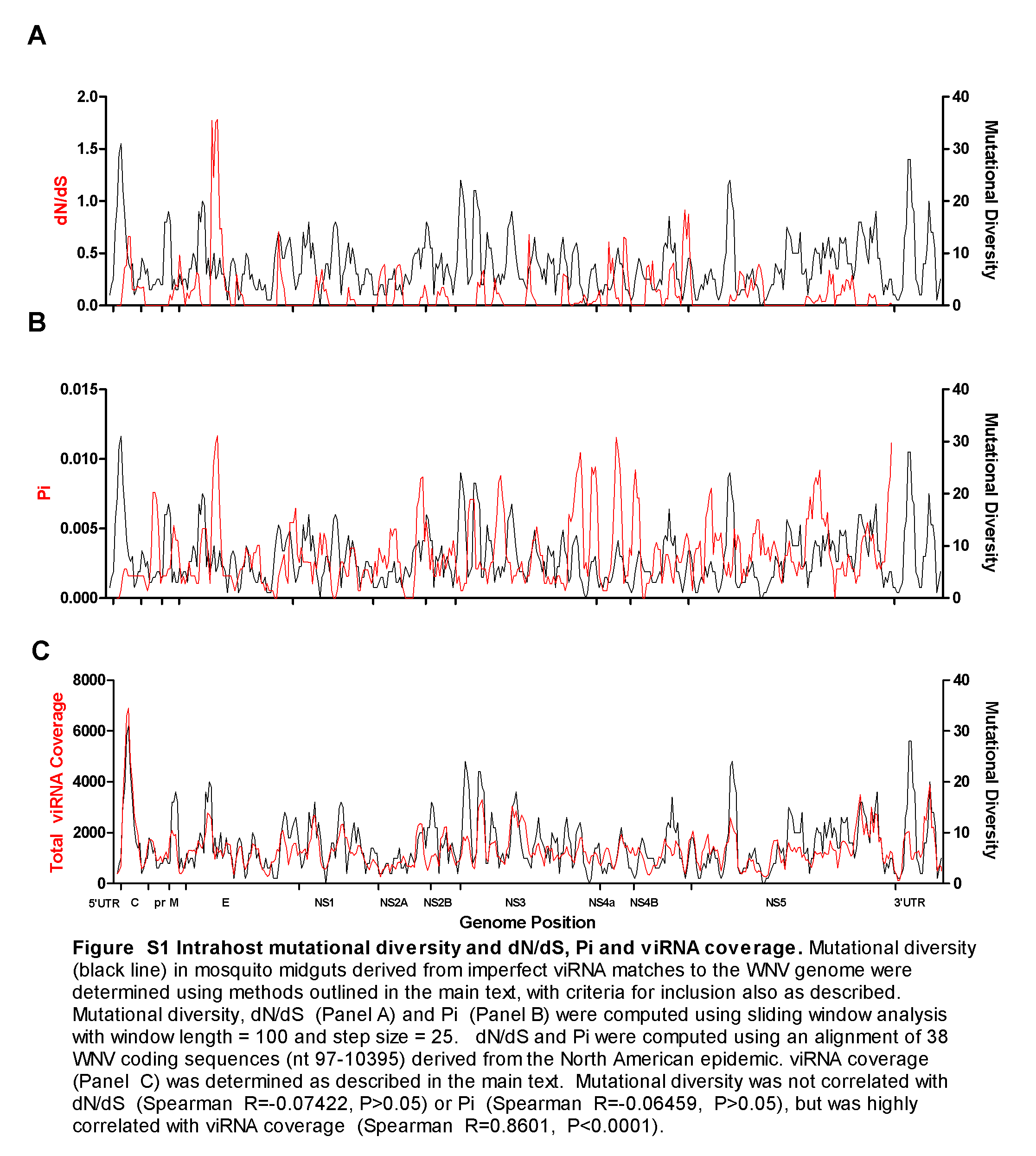

Supplement: Figure S1 — Intrahost mutational diversity and dN/dS, Pi and viRNA coverage. (1.73 MB TIF) [file ppat.1000502.s001.tif]
