# Supplementary material for: RNAi Targeting of West Nile Virus in Mosquito Midguts Promotes Virus Diversification
Source: PLoS Pathog. 2009 Jul 3;5(7):e1000502. doi: 10.1371/journal.ppat.1000502 (PMC2698148; doi:10.1371/journal.ppat.1000502)
Supplement: Table S1 — Genetic diversity and selection in cloned WNV. * An alignment of 38 WNV genomes obtained from the North American epidemic was used to generate interhost diversity values. # Undefined. Only nonsynonymous mutations were present in the alignment, yielding a denominator of zero for dN/dS calculation. (0.01 MB PDF) [file ppat.1000502.s002.pdf]

Table S1 Genetic diversity and selection in cloned WNV

	Genome Region Analyzed		
	ns5		3'UTR
Alignment	Percent Mutation (n)	dN/dS	Percent Mutation (n)
Day 7 Intra	0.0119 (16,761)	Und.	0 (6,440)
Day 14 Intra	0.00597 (16,761)	Und.	0.0454 (6,600)
Interhost*	0.110 (17,214)	0	0.114 (6,118)
