# Supplementary material for: RNAi Targeting of West Nile Virus in Mosquito Midguts Promotes Virus Diversification
Source: PLoS Pathog. 2009 Jul 3;5(7):e1000502. doi: 10.1371/journal.ppat.1000502 (PMC2698148; doi:10.1371/journal.ppat.1000502)
Supplement: Table S2 — Alignment of sRNA reads against viruses not used in these studies. * For all four sRNA libraries generated in these studies (WNV-infected and uninfected). (0.01 MB PDF) [file ppat.1000502.s003.pdf]

Table S2 Alignment of sRNA reads against viruses not used in these studies.

Virus	Accession Number	Length	Mismatches required for alignment: 0	1	2	Total*	Reads per kb
West Nile virus (Flavivirus)	AF404756	11,029	5,873	856	314	7,043	639
St. Louis Encephalitis virus (Flavivirus)	NC_007580	10,940	44	131	207	382	35
Powassan virus (Flavivirus)	AF311056	10,800	0	4	68	72	7
T'ho virus (Flavivirus)	EU879061	1,358	0	4	28	32	24
Culex Flavivirus (Flavivirus)	EU879060	10,837	0	1	27	28	3
Cell Fusing Agent virus (Flavivirus)	NC_001564	10,695	0	0	15	15	1
Eastern Equine Encephalitis virus (Alphavirus)	NC_003899	11,675	0	1	29	30	3
