# Supplementary material for: RNAi Targeting of West Nile Virus in Mosquito Midguts Promotes Virus Diversification
Source: PLoS Pathog. 2009 Jul 3;5(7):e1000502. doi: 10.1371/journal.ppat.1000502 (PMC2698148; doi:10.1371/journal.ppat.1000502)
Supplement: Text S1 — Supplemental text. (0.04 MB PDF) [file ppat.1000502.s004.pdf]

Supplement to Brackney et al.

Methods.

Analysis of Diversity and Selection.

To assess natural selection in the WNV populations within mosquito midguts, we computed dN/dS values for WNV sequences cloned from mosquito midguts at 7 and 14 days PI (Table S1). dN/dS was computed according to methods described elsewhere[2]. For comparison, an alignment of 38 complete WNV genome sequences obtained from the North American epidemic were aligned and dN/dS computed for the same regions cloned.

The accession numbers of the WNV genome sequences used are: FJ151394, NC_009942, EF657887, DQ666450, DQ666449, DQ666448, DQ211652, DQ211652, DQ164205, DQ164204, DQ164203, DQ164202, DQ164201, DQ164200, DQ164199, DQ164197, DQ164198, DQ164196, DQ164195, DQ164194, DQ164193, DQ164192, DQ164191, DQ164190, DQ164189, DQ164188, DQ164187, DQ164186, AY842931, AY660002, AY712947, AY712946, AY712946, AF404756, AF404755, AF404753, AF196835, AF260967.

To evaluate the possibility that differences in selective constraint led to the observed differences in intrahost genetic diversity (reported in the main text), we computed π (the mean nucleotide distance between sequences) and dN/dS along the complete WNV coding sequence using the alignment of 38 genomes described above. For these analyses

the window size was 100 nt and the step size was 25 nt. Mutational diversity was inferred from mismatching basecalls from sRNA reads aligning to the WNV genome as described in the main text, with mismatches with Illumina quality scores less than 30 discarded. Mutational diversity was then plotted using a sliding window as above. All analyses were performed using DnaSP[7], Microsoft Excel, PERL, R and GraphPad.

<u>viRNA specificity.</u>

To evaluate the possibility that other flaviviruses might be infecting our colony, we aligned all four (infected and uninfected) sRNA libraries against several irrelevant *Flavivirus* sequences and an *Alphavirus* sequence, using SOAP and matching criteria as described in the main text.

Results.

<u>Diversity and Selection.</u>

We observed approximately tenfold lower genetic diversity in the cloned WNV RNA than in our alignment of interhost WNV strains in both regions analyzed (Table S1). The interhost coding sequence had dN/dS = 0 because no nonsynonymous variation was detected in the region analyzed. Intrahost dN/dS values were undefined (i.e. infinity) at both 7 and 14 days because only nonsynonymous variation was detected.

Mutational diversity was inferred from mismatched bases in reads mapping to the WNV genome and plotted against dN/dS, π and the intensity of viRNA coverage (Figure S1). Inspection of the plots suggested, and statistical tests confirmed, that mutational diversity

was not correlated with either dN/dS or π, but was correlated with viRNA coverage. The most notable finding from this analysis was a weak signature of positive selection associated with the region of the E coding sequence that defines the WN02 genotype, which has been linked to reduced EIP in *Culex* mosquitoes and increased vectorial capacity[1,6].

viRNA Specificity.

sRNA libraries were aligned to genome sequences from several viruses not intentionally included in these studies (Table S2). More than tenfold more sRNA reads (obtained from all four libraries sequenced in this work) aligned to the WNV genome than to any other virus, including closely related agents such as SLE and those that might be expected to cryptically infect our colony (i.e. CFAV).

Discussion.

In previously published studies we demonstrated that intrahost genetic diversity is greater in mosquitoes than in birds, and that within these hosts the strong purifying selection that is characteristic of WNV populations in nature seems to be relaxed[2-4]. The analyses of synonymous and nonsynonymous variation reported here are consistent with either this interpretation (relaxed purifying selection within mosquitoes) or with positive selection, driven by RNAi, within hosts. In our current study, genetic variation in intrahost samples was low, but all detected mutations were nonsynonymous, supporting that purifying

selection may be relaxed in these hosts. In the context of our experimental design and aims, this was not particularly surprising: we infected mosquitoes with a homogeneous virus population orally, held them for 7 or 14 days EI, and then sampled sRNAs and virus genomes from the midguts. No virus dissemination or transmission was required in order for the virus to be sampled. Moreover, the peculiarities of the WNV transmission cycle that would seem to be most effective in imposing strong purifying selection were excluded from this particular experimental design. It is impossible to determine from the studies undertaken here whether the mutants sequenced by either method were from viable virus genomes or to assess their fitness.

Further, we failed to observe any correlation between mutational diversity estimated from sRNA reads mapping to the WNV genome and positive or negative selection or π. Again, this is not particularly surprising since none of the events thought to be important in imposing purifying selection on WNV (dissemination from the midgut, invasion of and release from salivary glands, infection of vertebrates, etc.) had occurred. The strong correlation with viRNA coverage shown in Figure S1 was expected because the data derive from the same set of data and are autocorrelated, i.e. the more reads covering a given position, the more likely a mutation is to be sampled. Therefore we independently ascertained mutation frequency by cloning and sequencing, as described in the main text. These results provide an alternative (or supplemental) interpretation of elevated dN/dS within mosquitoes, which is that RNAi is an agent of positive selection within hosts.

Finally we assessed the likelihood that previously undetected flaviviruses might be present in our colony by RT-PCR (reported in the main text), and by attempting to align sRNA libraries to irrelevant viruses. Very few reads mapped to anything other than the WNV genome, with the possible exception of SLEV and T'ho virus. Reads mapping to SLEV targeted a highly conserved portion of the 3'UTR. The T'ho virus genome is not yet complete, so it is difficult at present to generalize about which portions of the genome could be targeted. All other agents were poorly matched by sRNA reads. Combined with our RT-PCR data, these observations suggest that no other flaviviruses are cryptically infecting the mosquitoes used in these studies.

Reference List

1. Ebel, G. D., J. Carricaburu, D. Young, K. A. Bernard, and L. D. Kramer. 2004. Genetic and phenotypic variation of West Nile virus in New York, 2000-2003. Am J Trop Med Hyg 71:493-500.

2. Jerzak, G., K. A. Bernard, L. D. Kramer, and G. D. Ebel. 2005. Genetic variation in West Nile virus from naturally infected mosquitoes and birds suggests quasispecies structure and strong purifying selection. J Gen. Virol. 86:2175-2183.

3. Jerzak, G. V., K. Bernard, L. D. Kramer, P. Y. Shi, and G. D. Ebel. 2006. The West Nile virus mutant spectrum is host-dependant and a determinant of mortality in mice. Virology.

4. Jerzak, G. V. S., I. Brown, P. Y. Shi, L. D. Kramer, and G. D. Ebel. 2008. Genetic diversity and purifying selection in West Nile virus populations are maintained during host switching. Virology 374:256-260.

5. Moudy, R. M., A. Dupuis, G. D. Ebel, and L. D. Kramer. 2006. Displacement of the introduced genotype of West Nile virus in New York state. Am. J. Trop. Med. Hyg. 75:300.

6. Moudy, R. M., M. A. Meola, L. L. Morin, G. D. Ebel, and L. D. Kramer. 2007. A newly emergent genotype of West Nile virus is transmitted earlier and more efficiently by Culex mosquitoes. Am. J. Trop. Med. Hyg. 77:365-370.

7. Rozas, J., J. C. Sanchez-DelBarrio, X. Messeguer, and R. Rozas. 2003. DnaSP, DNA polymorphism analyses by the coalescent and other methods. Bioinformatics. 19:2496-2497.
